# Interplay between Catalyst Corrosion and Homogeneous Reactive Oxygen Species in Electrochemical Ozone Production

**DOI:** 10.1021/acscatal.4c01317

**Published:** 2024-04-18

**Authors:** Rayan Alaufey, Lingyan Zhao, Andrew Lindsay, Tana Siboonruang, Qin Wu, John A. Keith, Ezra Wood, Maureen Tang

**Affiliations:** † Department of Chemical and Biological Engineering, Drexel University, 3141 Chestnut Street, Philadelphia, Pennsylvania 19104, United States; ‡ Department of Chemical and Petroleum Engineering, University of Pittsburgh, 3700 O’Hara Street, Pittsburgh, Pennsylvania 15261, United States; § Department of Chemistry, Drexel University, 3141 Chestnut Street, Philadelphia, Pennsylvania 19104, United States; ∥ Center for Functional Nanomaterials, Brookhaven National Laboratory, Upton, New York 11973, United States

**Keywords:** ozone, electrocatalysis, corrosion, lattice oxygen, tin oxide, radicals

## Abstract

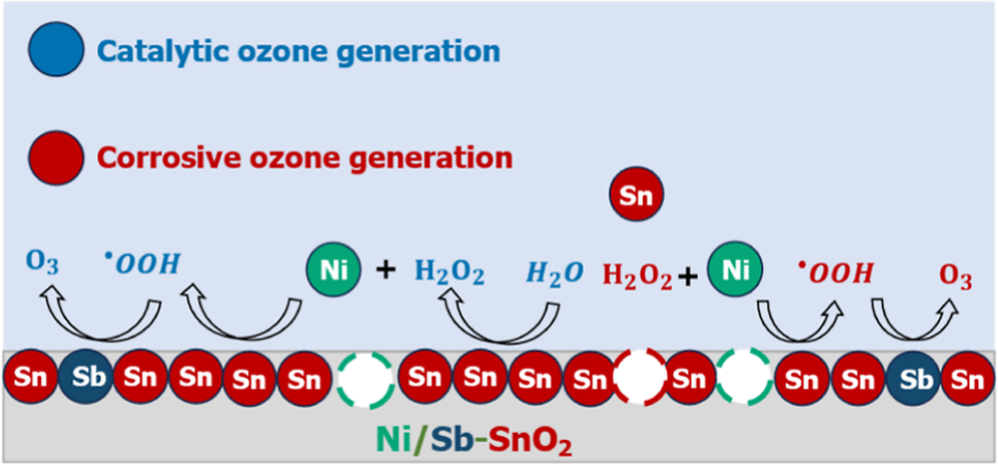

Electrochemical ozone production (EOP), a six-electron water oxidation reaction, offers promising avenues for creating value-added oxidants and disinfectants. However, progress in this field is slowed by a dearth of understanding of fundamental reaction mechanisms. In this work, we combine experimental electrochemistry, spectroscopic detection of reactive oxygen species (ROS), oxygen-anion chemical ionization mass spectrometry, and computational quantum chemistry calculations to determine a plausible reaction mechanism on nickel- and antimony-doped tin oxide (Ni/Sb–SnO_2_, NATO), one of the most selective EOP catalysts. Antimony doping is shown to increase the conductivity of the catalyst, leading to improved electrochemical performance. Spectroscopic analysis and electrochemical experiments combined with quantum chemistry predictions reveal that hydrogen peroxide (H_2_O_2_) is a critical reaction intermediate. We propose that leached Ni^4+^ cations catalyze hydrogen peroxide into solution phase hydroperoxyl radicals (^•^OOH); these radicals are subsequently oxidized to ozone. Isotopic product analysis shows that ozone is generated catalytically from water and corrosively from the catalyst oxide lattice without regeneration of lattice oxygens. Further quantum chemistry calculations and thermodynamic analysis suggest that the electrochemical corrosion of tin oxide itself might generate hydrogen peroxide, which is then catalyzed to ozone. The proposed pathways explain both the roles of dopants in NATO and its lack of stability. Our study interrogates the possibility that instability and electrochemical activity are intrinsically linked through the formation of ROS. In doing so, we provide the first mechanism for EOP that is consistent with computational and experimental results and highlight the central challenge of instability as a target for future research efforts.

## 1 Introduction

Electrochemical water treatment promises to address the pressing need for sustainable, efficient, and versatile solutions to global clean water scarcity.^1,2^ Generating disinfectants in situ can potentially lower treatment costs, and electrochemical processes are amenable to intermittent renewable electricity sources.^3,4^ Within the field of electrochemical water treatment, electrochemical ozone production (EOP, reaction 1) is particularly compelling.^5,6^ Ozone (O_3_) has been used as a disinfectant in various applications, including water purification and medical sterilization, and its ability to degrade pharmaceutical compounds and contaminants has been successfully demonstrated.^5−8^ Despite its promise, EOP requires electrocatalysts with improved stability and selectivity to become economically viable.^9−11^
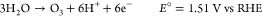
1
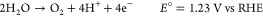
26-e^–^ EOP (eq 1) and 4-e^–^ OER (eq 2).

High selectivity for O_3_ is unexpected because the competing oxygen evolution reaction (OER), a four-electron process, is thermodynamically favored (Reaction 2). Only a few catalysts exhibit any selectivity for EOP.^7,12,13^ Of these, lead oxides (PbO_2_) are the most extensively researched.^8,14−16^ However, PbO_2_ has limited EOP selectivity and raises toxicity concerns, particularly for water treatment.^17,18^ While less studied, nickel and antimony-doped tin oxide (Ni/Sb–SnO_2_, NATO) is more promising based on toxicity and selectivity,^7,13^ yet is known to be relatively less stable.^18,19^ A comprehensive understanding of the reaction mechanism and the underlying cause of NATO instability would aid approaches to improved performance.

It is unclear how mechanistic studies of EOP on PbO_2_ apply to NATO, which exhibits significantly different electronic properties and reactivity, even though they share similar crystal structures and are composed of group (IV) post-transition metals.^20,21^ Unlike quasi-metallic PbO_2_, undoped SnO_2_ (TO) is a wide-bandgap semiconductor with poor electrical conductivity.^22−25^ Therefore, it is often n-type doped with antimony (Sb), which is known to increase its conductivity.^13,26^ While antimony-doped tin oxide (ATO, Sb–SnO_2_) can oxidize organic compounds and pollutants, neither undoped TO nor ATO generate O_3_.^18,27^ The introduction of nickel (Ni) as a co-dopant remarkably triggers EOP activity, even though the molar concentration of Ni is frequently less than 0.1%.^18,19^ The role of Ni in the catalyst is disputed and presents an interesting conundrum.^7,13,28^

Despite these differences, EOP on both PbO_2_ and NATO electrodes has typically been discussed in the literature in terms of the adsorbate evolution mechanism (AEM).^6,7,19,27,29−31^ In the AEM, water molecules adsorb to active sites on the surface of the catalyst, and O_3_ is generated through sequential electron–proton transfers involving surface-bound intermediates such as O* and OH*.^13,32−34^ However, additional evidence suggests that EOP also involves the lattice oxygen mechanism (LOM). LOM entails the direct coupling of lattice oxygens on the metal oxide surface.^15,35^ Recently, Jiang and co-workers used differential electrochemical mass spectrometry and ^18^O isotopically labeled water to show that the majority of generated O_3_ can be traced back to the catalyst oxide lattice.^35^ Similarly, Liu et al. showed that a Pb_3_O_4_ precatalyst can improve EOP performance; the precatalyst reconstructed to β-PbO_2_ (the tetragonal polymorph of PbO_2_) by exchanging its lattice oxygen with water. The reconstructed catalyst demonstrated enhanced EOP activity.^36^ Those findings are further supported by density functional theory (DFT) calculations, which suggested that O_3_ can be produced via LOM on a β-PbO_2_ (110) facet.^15,16^ LOM has been suggested to take place in other systems, including perovskite OER catalysts in alkaline conditions,^37−39^ as well rutile-type catalysts in acid.^40,41^ In contrast, the role of lattice oxygen in EOP on NATO electrodes has not been investigated.

The role of LOM on PbO_2_ motivates investigation into the role of lattice oxygen and its relation to instability on NATO. For LOM to be a truly catalytic process, lattice oxygens that evolve into either O_3_ or O_2_ must be exchanged with water. If lattice oxygen is not replenished with water-derived oxygen, the anode will be irreversibly corroded. Geiger and co-workers investigated the corrosion of ATO (EOP inactive) in acid and proposed that corrosion and OER activity are linked.^42^ EOP operates at even higher potentials and more corrosive conditions than OER.^19,20,31,42^ The possibility that the production of O_3_ directly from the irreversible consumption of lattice oxygens and metal cations on NATO has not been explicitly addressed. Consequently, the interplay between anode corrosion and lattice oxygen activity for EOP deserves investigation.

Finally, previous work has suggested that homogeneous reactive oxygen species (ROS) act as intermediates in EOP. Our group showed that increasing the thickness of NATO electrodes increased both reaction activity and selectivity, which cannot be explained without invoking transport of solution-phase intermediates.^28^ Work by Ding et al. and Zhang et al. revealed that NATO catalysts generate hydroxyl radicals (^•^OH).^43,44^ Lansing et al. showed that quenching ^•^OH had a minimal effect on EOP from NATO electrodes, while quenching hydroperoxyl radicals (^•^OOH) significantly decreased it.^32^ These findings diverge from the conventional perspectives of both AEM and LOM, which typically assume the involvement of only surface-adsorbed intermediates. If solution-phase ^•^OOH are intermediates in EOP, the selectivity of the rection on NATO electrodes can be viewed as a competition between surface-mediated OER and homogeneous ROS production, leading to O_3_ generation. More work is needed to understand the role of ROS and other solution-phase intermediates in EOP.

This work investigates these open questions using a combined experimental and computational approach to consolidate findings into one comprehensive mechanism. We first demonstrate the instability of NATO electrocatalysts. We investigate the presence of ROS and identify critical reaction intermediates through the spectroscopic detection of radical-selective chemical probes at different applied potentials. Lattice oxygen participation and catalyst corrosion are investigated using oxygen-anion chemical ionization mass spectrometry (CIMS). The thermodynamic feasibility of elementary steps and the nature of dissolved cations are investigated with computational quantum chemistry. Together, these results allow the development of a comprehensive mechanism for EOP on NATO electrodes.

## 2 Experimental Methods

### 2.1 Chemicals

All chemicals were purchased and used as received. SnCl_4_–5H_2_O (98%), SbCl_3_ (>99%), NiCl_2_–6H_2_O (98%), titanium foil (0.127 mm, 99.99%), H_2_SO_4_ (99.99), and benzoic acid (≥99.5%) were from Sigma-Aldrich. Oxalic acid (10% w/v) was from Beantown Chemical, ethanol (200 proof, anhydrous) was from Deacon Laboratories, dihydroethidium (DHE) was from AnaSpec, and ^18^O isotopically labeled water was from Medical Isotopes Inc. (98.5% ^18^O).

### 2.2 Electrode Synthesis

Methods were adapted and modified from Lansing et al.^45^ NATO electrodes were synthesized using SnCl_4_–5H_2_O, SbCl_3_, NiCl_2_–6H_2_O, and C_2_H_4_OH. A catalyst precursor solution in 5 mL of pure ethanol was prepared with a precursor mole ratio of (1:16:250/Ni/Sb/Sn) for all parts except cyclic voltammetry, in which a ratio of (1:3:96/Ni/Sb/Sn) was used. Ti foil was cut into 0.5 × 0.5 cm^2^ substrates. The substrates were then chemically etched by boiling in 50 mL of oxalic acid for 30 min. Etched substrates were washed and sonicated with Millipore water and immediately preheated to 85 °C on a silicon carrier wafer to be used for electrode synthesis. The precursor solution was evenly drop-cast on each substrate. The samples were held at 65 °C for three min to allow ethanol to evaporate, and then they were sintered at 450 °C for five min inside a muffle furnace. After removal from the furnace, the samples were cooled in air and turned over to the opposite side. The procedure was repeated nine more times for a total of ten applications (5 applications on each side). On the final application, the samples remained in the muffle furnace at 450 °C for 60 min. A Ti wire (0.125 mm thick) was spot-welded to one side of the electrode. The total change in the substrate mass after sintering was 5.26 ± 0.27 mg. TO and ATO electrodes were prepared following the same procedure.

### 2.3 Catalyst Characterization

Scanning electron microscopy (SEM) images of the electrodes were taken by using a Zeiss Supra 50VP at a working distance of 9 mm and an accelerating working voltage of 5.00 kV. X-ray photoemission spectroscopy (XPS) measurements were performed with a Versa Probe 5000 spectrometer (Physical Electronics Inc., USA) with monochromatic Al Kα radiation and a beam setting of 200 μm with 25 W and 15 kV. CasaXPS was used for peak fitting.^46^ Adventitious carbon’s C 1s peak at 284.8 eV was used for charge correction. X-ray diffraction measurements were performed using a Rigaku Miniflex X-ray diffractometer (XRD) in the Bragg–Brentano geometry with a Cu Kα filter (λ = 1.54056 Å). Catalyst conductivity was assessed by depositing the films onto quartz substrates, employing the same preparation conditions as those previously described. Four-point probe measurements were conducted using an Ossila T2001A3 system at room temperature with a current under a 1 mA current range.

### 2.4 Electrochemical Testing and Ozone Measurements

Methods were adapted from Lees et al. and Wang et al.^28,29^ NATO electrocatalysts were used as working electrodes; a platinum wire was used as a counter electrode, and a BASI Ag/AgCl in 3.0 M KCl was used as a reference electrode. For electrochemical testing, the electrodes were placed in a 4.5 mL quartz cuvette with an airtight seal. The electrodes were connected to a Biologic potentiostat. A fresh electrolyte and an electrode were used for each test. For flux and selectivity measurements, a constant potential of 2.70 V vs RHE was applied for 1 min. A PerkinElmer Lambda 35 UV–vis spectrometer was used to measure the absorbance of O_3_ at 258 nm. The spectrometer absorbance background was measured immediately before the start of each test. The concentration was determined from absorbance via Beer’s law with a molar extinction coefficient of 3000 M^–1^ cm^–1^.^47,48^ The molar electrode flux and current efficiency (CE) were then calculated from the following equations
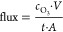
3
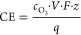
4where *c*_O_3__ is the concentration of O_3_ calculated from absorbance, *V* is the volume of the cell (4.5 mL), *t* is the electrolysis time (1 min), *A* is the geometric area of the electrode (0.5 cm^–2^), *F* is Faraday’s constant, *z* is the number of electrons (6), and *q* is the total charge generated during electrolysis.

### 2.5 Free Radical Detection

^•^OH radicals were detected via their reaction with benzoic acid to form hydroxybenzoic acids. Constant potential electrolysis at 2.70 V vs RHE was carried out in a 10 mL solution of 1.5 mM BA and 0.5 M H_2_SO_4_. Post electrolysis, the pH of the solution was titrated to 5.5 using NaOH to increase the emission intensity of the products, which is dependent on the pH of the solution.^49^ Product concentrations were quantified at an excitation wavelength of 320 nm and an emission wavelength of 440 nm by using a Shimadzu RF-6000 fluorescence spectrometer. ^•^OOH radicals were detected via their reaction with DHE to form 2-hydroxy ethidium (2-OH^+^). A 30 μM stock solution of DHE was first prepared. 10 μL of the stock solution was directly injected on top of the working electrode 50 s into 1 min electrolysis. The absorbance of 2-OH^+^ near 440 nm was measured using a PerkinElmer Lambda 35 UV–visible spectrophotometer.

### 2.6 Chemical Ionization Mass Spectrometry

O_3_ isotopologues were measured via oxygen anion CIMS, in which they are ultimately detected as either CO_3_^–^ or C(^18^O)O_2_^–^. The ion chemistry is described in Section 3.3. The evolved O_3_ from the electrochemical cell was sampled, along with 0.1 standard liters per minute (SLPM) of room air, into 20 cm of 0.40 cm ID fluorinated ethylene propylene (FEP) tubing. This flow was diluted with 1.9 SLPM of dry nitrogen (Airgas, industrial grade), flowed through another 60 cm of FEP tubing, and sampled through a 75 μm stainless steel orifice into a laboratory-built ion–molecule reactor (IMR) held at 80 mbar and internally coated with FEP.^50^ In the IMR, the sampled flow is mixed with 2 SLPM of air containing gas-phase O_2_^–^ ions prepared by exposure of zero air (Airgas, ultrazero air grade) to alpha radiation from a commercial ^210^Po-based ionizer (NRD P-2021, 10 mCi). Most of the flow from the IMR continues toward a scroll pump (Agilent IDP-7) choked to maintain a constant IMR pressure of 80 mbar. A smaller portion of the flow enters the mass spectrometer (API-ToF, Aerodyne Research, Inc./TofWerks) via a second critical orifice. The mass spectrometer comprises two consecutive differentially pumped transfer stages in which neutral gases are pumped away and ions are guided toward the next stage via RF-only (nonmass filtering) segmented quadrupoles. The ions finally enter a time-of-flight region (resolving power 5500) and are detected with a microchannel plate detector. A more detailed description of the ion chemistry and the instrument can be found in works by Novak et al.^51^ and Bertram et al.^52^

The main ions of concern are the “light” and “heavy” CO_3_^–^ peaks at *m*/*z* 59.9847 and 61.9847, corresponding to carbonate ions containing zero or one ^18^O. High-resolution fitting procedures (TofWare software, Tofwerk) were used to determine the contribution of the actual ions of interest at their exact mass-to-charge ratio. The voltage settings used for the quadrupoles and ion optics were optimized while sampling a constant concentration of *O*_3_(*g*) produced by photolysis of *O*_2_(*g*) (2B Tech model 306). The large sample dilution resulting from the sampled room air and the 1.9 SLPM of added nitrogen reduced the O_3_ concentrations to within the working range of the instrument by ensuring that the reagent O_2_^–^ ions remained in excess. The CO_3_^–^ and C(^18^O)O_2_^–^ signals observed when only room air was sampled (prior to commencing the electrolysis) were 1900 and 220 counts s^–1^, respectively, which are 8 and 300 times lower than the values observed when evolved O_3_ was sampled from the cell. Much smaller amounts of the “bare” O_3_^–^ peaks were also observed and served as confirmation that three of the four O_3_ isotopologues were present (see Section 3.3).

### 2.7 Computational Quantum Chemistry Methods

Kohn–Sham DFT was performed on a 2 × 2 × 4 SnO_2_ (110) surface with the bottom two layers fixed to the bulk structure and the upper two layers relaxed to model the surface reaction. All surface geometries were relaxed until the energy difference between steps was less than 1 meV. All surface gas phase electronic energies were calculated using the PBE exchange–correlation functional^53^ and projector augmented wave pseudopotentials^54,55^ with spin polarization enabled in the GPU port^56,57^ of the Vienna Ab initio Simulation Package (VASP) 5.4.4.^58,59^ For all surface calculations, we used an energy cutoff of 450 eV and 4 × 4 × 1 Monkhorst–Pack grid sampling of *k*-points. For molecular species modeled with VASP, the molecule was placed in a 15 × 15 × 15 Å simulation box with a gamma point *k*-point sampling. At least one explicit water molecule was used on each surface model to account for explicit solvent interactions and/or interactions with coadsorbed OH* and H* intermediates that arose from water dissociation.

Standard PBE functionals are well-known to have challenges accurately calculating electronic energies of molecular oxygen and other molecular radicals, so gas phase calculations for lone molecules were performed with the ORCA 4.2.0 code^60,61^ using the hybrid DFT functional B3LYP^62−64^ and D3 dispersion model with Becke-Johnson damping.^65,66^ The molecular geometries were first optimized using the Def2-SVP basis set^67^ in the gas phase, with a vibrational frequency calculation to validate that structures were at minima on the potential energy surface. Free energy contributions were calculated based on standard ideal gas, rigid rotor, and harmonic oscillator approximations. Higher-accuracy single-point gas phase energies were obtained using the Def2-TZVP basis set^67^ with the same geometry. The SMD continuum solvent model^68^ with default water parameters were used to describe the aqueous environment of molecular intermediates in a homogeneous solution using the same basis set as single-point energy calculations. Additional technical details are presented in the Supporting Information regarding calculations of oxidation potentials relevant for evaluating corrosion mechanisms.

## 3 Discussion and Results

### 3.1 Catalyst Performance and Characterization

**Figure 1:**
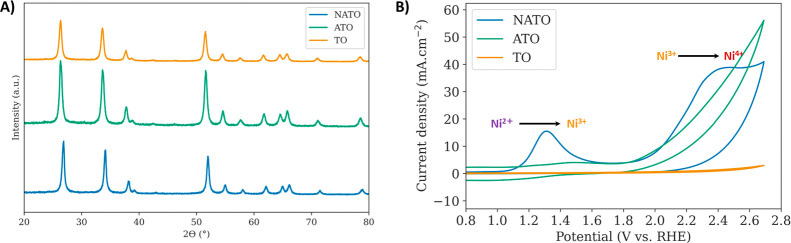
(A) Powder XRD patterns of TO (EOP inactive), ATO (EOP inactive), and NATO (EOP active). (B) Cyclic voltammetry of TO (EOP inactive), ATO (EOP inactive), and NATO (EOP active) at a scan rate of 75 mV s^–1^

Figure 1A shows XRD patterns for NATO, ATO, and TO. All three materials exhibited the rutile structure characteristic of SnO_2_.^69^ There were no observable signs of segregation due to additional crystalline phases attributable to the presence of Ni or Sb. It is worth noting that the amount of Ni in the precursor is lower than the detection limit of separate crystalline phases by XRD, which is typically around 1–2% by volume.^70,71^ Consequently, there is a possibility of undetectable crystalline secondary dopant phases in the catalyst. Furthermore, segregation could have occurred in the amorphous phase of the catalyst.

**Figure 2:**
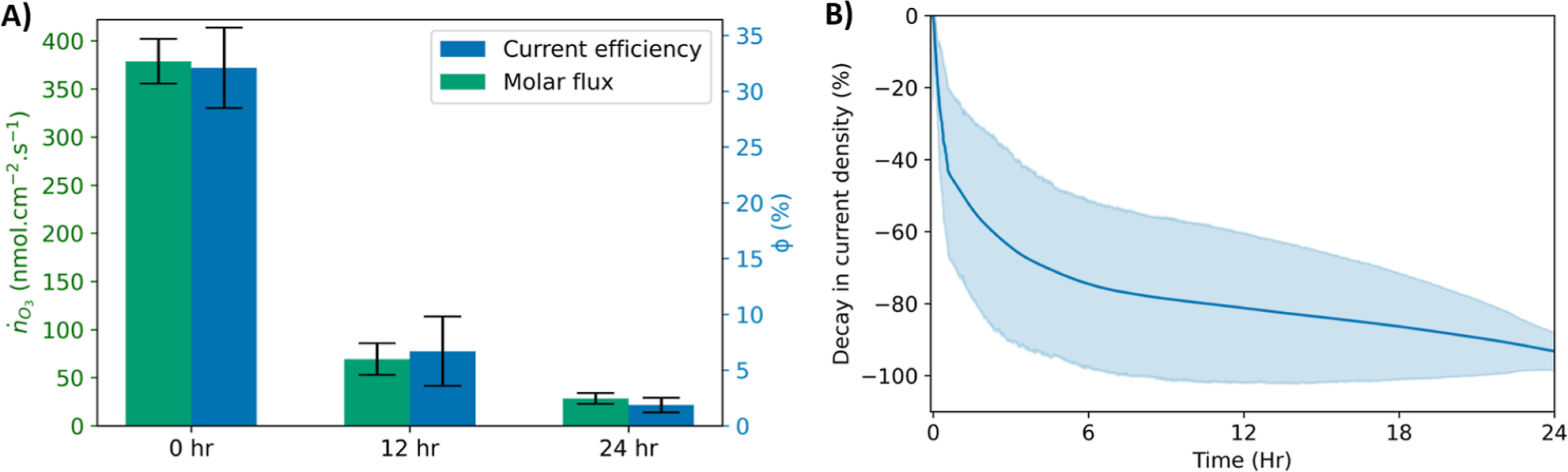
(A) Average O_3_ molar flux and current efficiency sampled from 1 min at three different points during the test. Three trials are shown. (B) Decay in current density for NATO during constant potential electrolysis in 0.5 M H_2_SO_4_. Solid line represents the average of three trials, and the shaded region represents the standard error.

Table 1 shows the crystallinity, lattice parameters, and electrical conductivity of all catalysts. While doping with Ni and Sb caused a slight contraction in the unit cell volume (likely due to the low doping ratio), no clear trends were observed in the crystallinity values. Electrical conductivity measurements revealed a notable increase when the catalyst was doped with Sb, while Ni doping had a minimal impact. This observation is consistent with the literature, which has shown that Sb donates an electron to tin oxide, leading to increased conductivity.^22,23,72,73^ Due to its lower conductivity, the total current density generated by TO was significantly lower than that of ATO and NATO as shown in the CVs in Figure 2B. We reemphasize that only NATO is EOP active.

**Table 1:** Lattice Parameters, Crystallite Size, and Electrical Conductivity

catalyst	*a* (Å)	*c* (Å)	average crystallite size (nm)	electrical conductivity (s/m)
TO	4.76	3.20	21.1	5.9
ATO	4.75	3.19	20.5	1.1 × 10^2^
NATO	4.71	3.17	23.7	1.2 × 10^2^

Additionally, the first scan for NATO exhibits two distinct peaks absent in the CV of the Ni-free ATO control. These peaks, at around 1.4 and 2.3 V, might correspond to the sequential oxidation of Ni^2+^ to Ni^3+^ and Ni^3+^ to Ni^4+^, respectively. Notably, these Ni-related oxidation features disappear in subsequent scans of NATO (Figure S2), suggesting that Ni cations leach out or corrode from the catalyst during oxidation, consistent with Pourbaix diagram predictions for Ni.^74^

Figure 2A displays the 1 min average molar O_3_ flux and current efficiency measured at three different time points: the beginning of the test (0 h), halfway through (12 h), and at the end (24 h). NATO electrodes’ ability to generate O_3_ deteriorates over time, with the average flux declining from  at the start of the test to 28  at the end and the current efficiency drops from 32 to 1.9%. The amounts of O_3_ generated by the electrodes as well as current efficiencies are consistent with previous reports.^7,28,29,45^Figure 2B shows the current density decay of NATO electrodes during potentiostatic electrolysis in 0.5 M H_2_SO_4_ over 24 h. The current density decays by 80–100% during the test period, demonstrating the instability of the NATO electrodes. These results are consistent with prior work by Sandin and co-workers showing that all elements initially present in NATO leach during EOP, leading to the deactivation of the electrode.^19^

**Figure 3:**
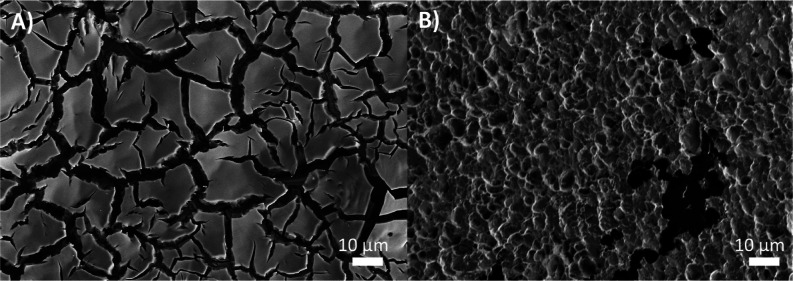
Representative SEM images of (A) fresh electrodes and (B) used electrodes (24 h). Black spots on the used electrode are attributed to carbon contamination.

**Figure 4:**
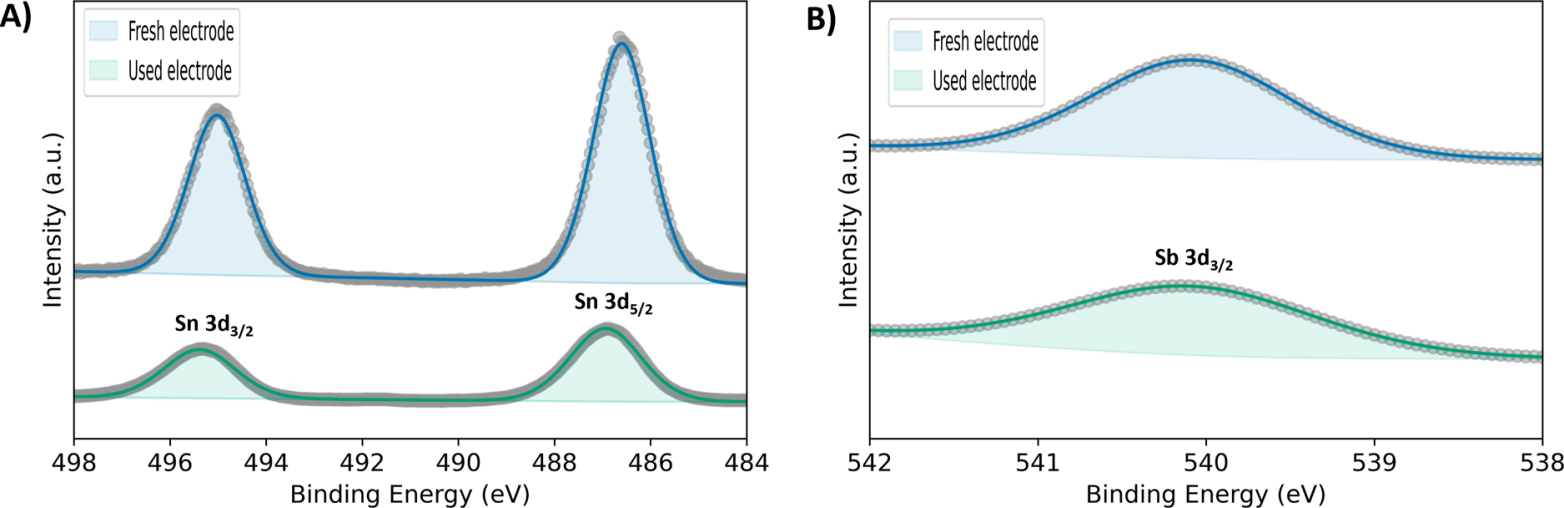
XPS spectra of fresh and used electrodes (24 h) of (A) Sn 3d region and (B) Sb 3d_3/2_ region.

SEM images of fresh and used (24 h) electrodes are shown in Figure 3. The cracked-mud morphology exhibited by fresh NATO electrodes is lost post-electrolysis, and used electrodes display a thinner film that shows clear signs of corrosion. Figure 4 shows the XPS spectra of the Sn 3d region for a fresh and used NATO electrodes (24 h). The orbital energy in both electrodes indicates that Sn is present mainly as Sn^4+^ but does not allow for quantitative discrimination between Sn^2+^ and Sn^4+^. The binding energy of the Sn 3d orbitals exhibits a blue shift post-electrolysis, possibly due to the oxidation of some residual Sn^2+^ to Sn^4+^. The Sb 3d_5/2_ and the O 1s peaks overlap. Therefore, the less intense Sb 3d_3/2_ peak was used to monitor the change in the amount of Sb. Figure 4B shows that Sb was present in fresh and used electrodes, however; due to similar peak positions of Sb^5+^ and Sb^3+^, distinguishing between the two oxidation states with certainty is challenging.^75^ Ni could not be detected on the surface of the electrode before or after electrolysis as shown in S5, which is consistent with the literature.^7,29,76^ Survey spectra of the fresh and used electrodes are also shown in S4. Given that used electrodes were capable of generating a small amount O_3_ after 24 h, it is reasonable to assume that a portion of all active elements remained present in the system. Together, Figures 2–4 demonstrate the corrosion of NATO electrodes during EOP, consistent with the literature.

### 3.2 ROS Detection and the Mechanism of EOP

To understand how O_3_ is generated on NATO electrodes and the role of Ni in the catalyst, we employed selective chemical probes to detect ^•^OH and ^•^OOH which have previously been linked to EOP on NATO electrodes.^7,77^ Since Ni-free ATO is EOP inactive, it was employed as a control electrode to distinguish radicals that are uniquely present in EOP from side-products.

**Figure 5:**
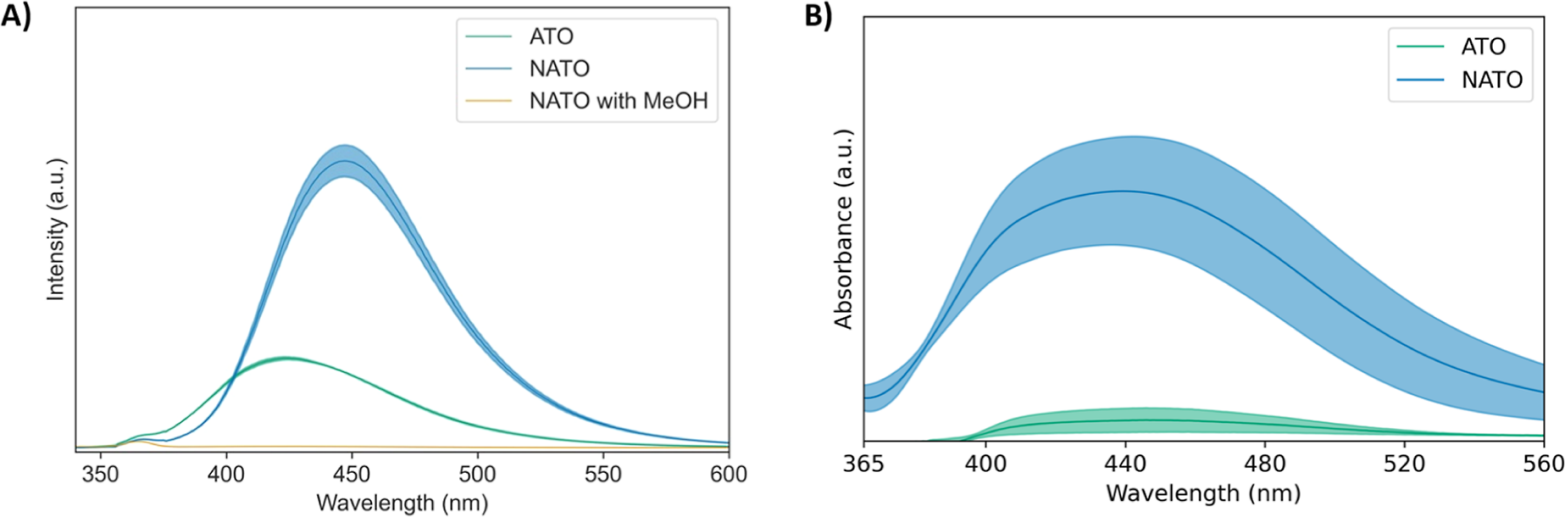
(A) Emission spectra of benzoic acid products post-electrolysis on NATO in blue (EOP active), ATO in green (EOP inactive), and NATO with methanol (MeOH) in yellow. (B) Absorbance spectra of DHE products post-electrolysis in the visible region with NATO in blue (EOP active) and ATO in green (EOP inactive). Solid line represents the average of three trials, and the shaded region represents the standard error.

Figure 5A shows that both NATO (EOP active) and ATO (EOP inactive) generate ^•^OH at 2.70 V, consistent with the literature.^7,43,44,77^^•^OH radicals were detected via their selective reaction with benzoic acid to generate hydroxybenzoic acids. Importantly, benzoic acid should be selective to ^•^OH because the presence of the carboxylic group in benzoic acid deactivates the electrophilic substitution reaction by O_3_.^83^ As shown in Figure 5A, the products of this reaction are detected via their fluorescence.^49,78,79^ The redshift in the emission peak of NATO (blue curve) may be due to an inner-filter effect caused by the increased concentration of hydroxybenzoic acid.^80^ To verify that hydroxybenzoic acid was formed selectively from homogeneous chemical hydroxylation, rather than direct electro-oxidation of benzoic acid on the surface, methanol was added to the reaction mixture pre-electrolysis. As shown in Figure 5A (yellow spectrum), ^•^OH radicals were not detected on NATO electrodes in the presence of the radical scavenger,^81,82^ further confirming the formation of solution-phase ^•^OH.

The absorbance spectra of 2-hydroxyethidium in Figure 5B shows that ^•^OOH radicals form on NATO (EOP active) but not on ATO (EOP inactive) electrodes at 2.70 V, suggesting that ^•^OOH radicals are uniquely involved in EOP, consistent with prior work.^32^ Notably, 2-hydroxyethidium is the selective red product produced from the reaction between colorless DHE and ^•^OOH.^84−87^ Prior work on NATO showed that quenching ^•^OH had a minimal impact on O_3_ production, whereas quenching ^•^OOH significantly reduced it.^32^ Combined with the findings in this study, our results suggest that ^•^OOH radicals are a reaction intermediate while ^•^OH radicals are a side product.

**Figure 6:**
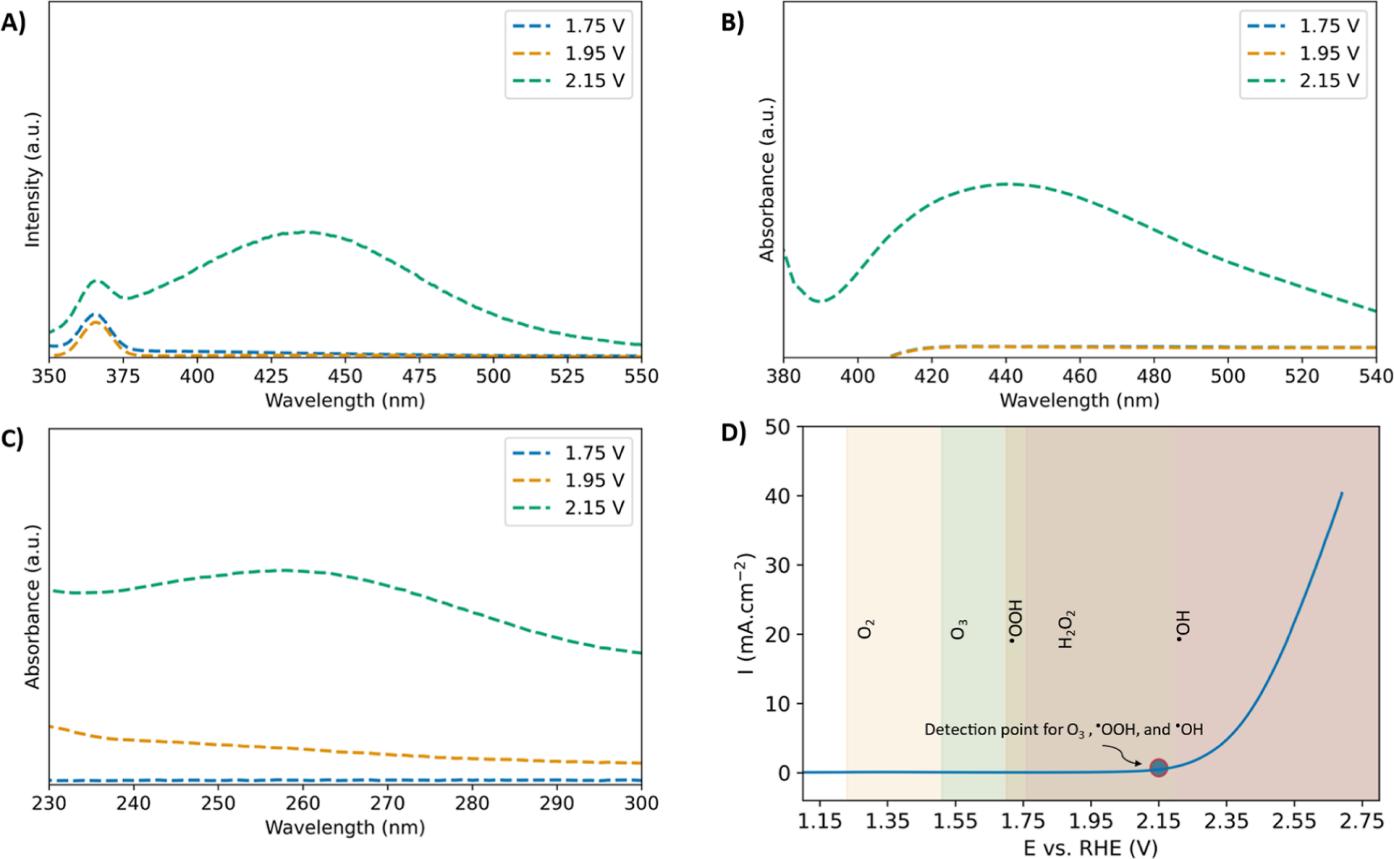
Effect of potential on (A) Emission spectra of benzoic acid (^•^OH probe). (B) Absorbance spectra of 2-hydroxyethidium (^•^OOH probe). (C) O_3_ absorbance spectra. (D) LSV of NATO electrodes at 75 mV s^–1^ showing the thermodynamic standard potentials for different chemical species generated electrochemically from water with the experimental detection point of O_3_, ^•^OH, and ^•^OOH.

We next varied the applied potential from 1.75 to 2.15 V to determine the onset of radicals and O_3_. Despite their different roles in EOP, ^•^OOH and ^•^OH emerged at the same potential as that of O_3_, as shown in Figure 6. The simultaneous appearance of all species at 2.15 V indicates an intrinsic relationship in the generation mechanism. Therefore, understanding the formation pathway of one species might provide valuable insights into the generation of all three. Importantly, indirect generation of radicals from O_3_ (due to its decay) requires the presence of hydroxide initiators (OH^–^), and is therefore minimal in 0.5 M H_2_SO_4_.^79,83^Table 2 summarizes the findings in this section.

**Table 2:** Summary of ROS Studies

species	NATO	ATO	addition of quenchers	detection potential (V)
^•^OH	detected	detected	no effect on O_3_^32^	2.15
^•^OOH	detected	not detected	negatively impacts O_3_^32^	2.15
O_3_	detected	not detected	N/A	2.15

Interestingly, direct electro-oxidation of water to ^•^OH radicals is not thermodynamically feasible at 2.15 V. Theoretical studies suggest that ^•^OH radicals can be generated by oxidizing water through a one-electron process.^88,89^ The thermodynamic potential of this reaction is most frequently cited as 2.73 V, which closely corresponds to our quantum chemistry predictions discussed in S5, although values as low as 2.20 V have been reported.^88,89^ If 2.20 V is taken as the true potential for the reaction, then it provides a reasonable explanation for the generation of ^•^OH at 2.70 V. However, it cannot explain the presence of ^•^OH on NATO at 2.15 V. Therefore, ^•^OH radicals are unlikely to be directly generated from water.

Alternatively, ^•^OH could be chemically produced from hydrogen peroxide (H_2_O_2_), which could be first generated through a two-electron oxidation reaction^88,90^
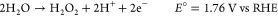
5

In the presence of dissolved transition metal cations (such as Ni), pseudo-Fenton reactions can decompose H_2_O_2_ to radicals, explaining the production of ^•^OH on NATO electrodes at 2.15 V.^2,91−95^ The CV presented in Figure 1B suggests that leached Ni is present as a mixture of Ni^2+^, Ni^3+^, and Ni^4+^. These cations can facilitate pseudo-Fenton reactions in our system. We used computational quantum chemistry modeling to verify the feasibility of this hypothesis by calculating the energy of possible pseudo-Fenton steps between H_2_O_2_ and different Ni cations. We assumed that dissolved Ni cations predominantly exist as Ni(OH)_*n*_(H_2_O)_4*n*_ species in solution where *n* = 2, 3, and 4 corresponding to the oxidation state of Ni. The free energies of all possible reactions are discussed in S2 Supporting Information. According to our calculations, ^•^OH radicals and Ni^3+^ cations are effectively isoenergetic with Ni^2+^ cations and H_2_O_2_, leading to a slightly negative free energy of formation for the reaction
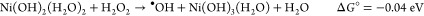
6

Therefore, our hypothesis is thermodynamically viable, and the generation of ^•^OH radicals in our system can be used to deduce that H_2_O_2_ is transiently present.

Because ^•^OOH radicals emerge at the same potential as ^•^OH, they are also likely to be produced from H_2_O_2_, in contrast to direct three-electron oxidation from water, which is further discussed in S4 Supporting Information. The simultaneous appearance of both radicals suggests that both originate from the same source. Furthermore, our quantum chemistry calculations show that the pseudo-Fenton reaction between H_2_O_2_ and dissolved Ni^4+^ cations can lead to the generation of ^•^OOH. The reduction of an Ni^4+^ complex to an Ni^3+^complex is significantly downhill, making it a viable route for the generation of ^•^OOH
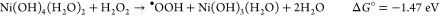
7

Our analysis presented above suggests that the main role of Ni in NATO is facilitating the production of ^•^OOH radicals via the reaction between leached Ni^4+^cations and H_2_O_2_. However, the amount of O_3_ that NATO is capable of producing is far greater than the undetectable amount of Ni present in the catalyst. Therefore, Ni^4+^ must be regenerated to maintain O_3_ production. Data in S2 Supporting Information show that the regeneration of Ni^4+^homogeneously via pseudo-Fenton processes is thermodynamically prohibited. Therefore, we hypothesize that Ni^3+^and Ni^2+^ can diffuse back to the anode where they are re-oxidized to Ni^4+^. This allows for the continuous production of ^•^OOH and subsequently, sustainable O_3_ generation. Similar mechanisms advocating for both the presence of Ni^4+^ and homogeneous activity/regeneration cycles of dissolved 4+ cations have been suggested for transition metal catalysts in OER.^96−101^

Finally, once ^•^OOH radicals are formed, they must be oxidized to O_3_. Many potential reactions between ^•^OOH and adsorbed species can lead to the formation of O_3_ while satisfying experimental observations. One possibility is the reaction with adsorbed oxygen (O*) which is expected to be present under reaction conditions. Our calculations on the SnO_2_ (110) surface found a moderately uphill electrochemical potential of 0.72 V for this reaction, suggesting that the process is thermodynamically viable at operating conditions.
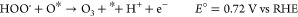
8

The detailed modeling is reported in Scheme S2 with additional benchmark calculations using different computational methods shown in Tables S4. Additionally, the free energies of all reactions discussed in this section (both viable and prohibited) are calculated in parts S4 and S2.

Despite strong circumstantial evidence of its presence and activity, we were unable to detect H_2_O_2_ directly. Although theoretical predictions have repeatedly proposed it can be generated anodically,^88,90^ experiments have been unable to prevent its rapid decomposition without a carbonate/bicarbonate supporting electrolyte.^102,103^ Furthermore, H_2_O_2_ in small amounts has a quenching effect on O_3_.^104,105^ Therefore, we attribute the inability to detect H_2_O_2_ to its rapid conversion to radicals, O_3_, and O_2_.

In summary, our ROS experimental results in combination with quantum chemistry calculations indicate that the mechanism of the EOP on NATO can be divided into three steps. First, H_2_O_2_ is generated from water oxidation (Reaction 5). Second, H_2_O_2_ is homogeneously oxidized by Ni^4+^ to generate ^•^OOH (Reaction 7). This step can be sustained only if Ni^4+^ is electrochemically re-generated. Finally, an oxidation reaction converts ^•^OOH radicals to the O_3_ (Reaction 8). We note that this analysis does not preclude other pathways that we have not yet investigated from forming radicals and O_3_ as well.

### 3.3 Oxygen Anion CIMS and Anodic Corrosion

With the understanding that ROS and H_2_O_2_ play an active role in EOP, we revisit electrode corrosion. Typical post-electrolysis characterization cannot determine if corrosion and lattice oxygen participation are linked or if catalyst loss simply occurs in parallel to O_3_ formation. To investigate this link, the generation of O_3_ in ^18^O-labeled water was monitored over 18 h using oxygen anion CIMS. By analyzing the isotopic composition of the generated gaseous O_3_, it is possible to identify whether oxygen atoms originate from the oxide lattice or water.

Using CIMS, we can detect O_3_ in two different ways. Mainly, sampled O_3_ can react to form carbonate anions (CO_3_^–^) following Reactions 9 and 10, all occurring in the gas-phase in the CIMS IMR
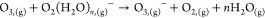
9
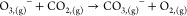
10

As discussed in Section 2.6, the oxygen anion reagent is prepared by the exposure of zero air to alpha radiation generated by a ^210^Po-based ionizer. The sources of H_2_O(g) and C*O*_2_(*g*) were the sampled indoor laboratory air. The value of the integer *n* cannot be determined based on the observed mass spectrum but is likely a combination of 0, 1, and 2. Notably, only a single, terminal oxygen atom from O_3_ is transferred to CO_2_ and present in the detected carbonate ion. As a result, two carbonate signals are expected: “light” C(^16^O)_3_^–^ at nominal *m*/*z* 60 and “heavy” C(^16^O)_2_^18^O^–^ at nominal *m*/*z* 62. The ratio of heavy carbonate/light carbonate thus indicates the ratio of terminal ^18^O to terminal ^16^O in the generated O_3_ isotopologues.

**Figure 7:**
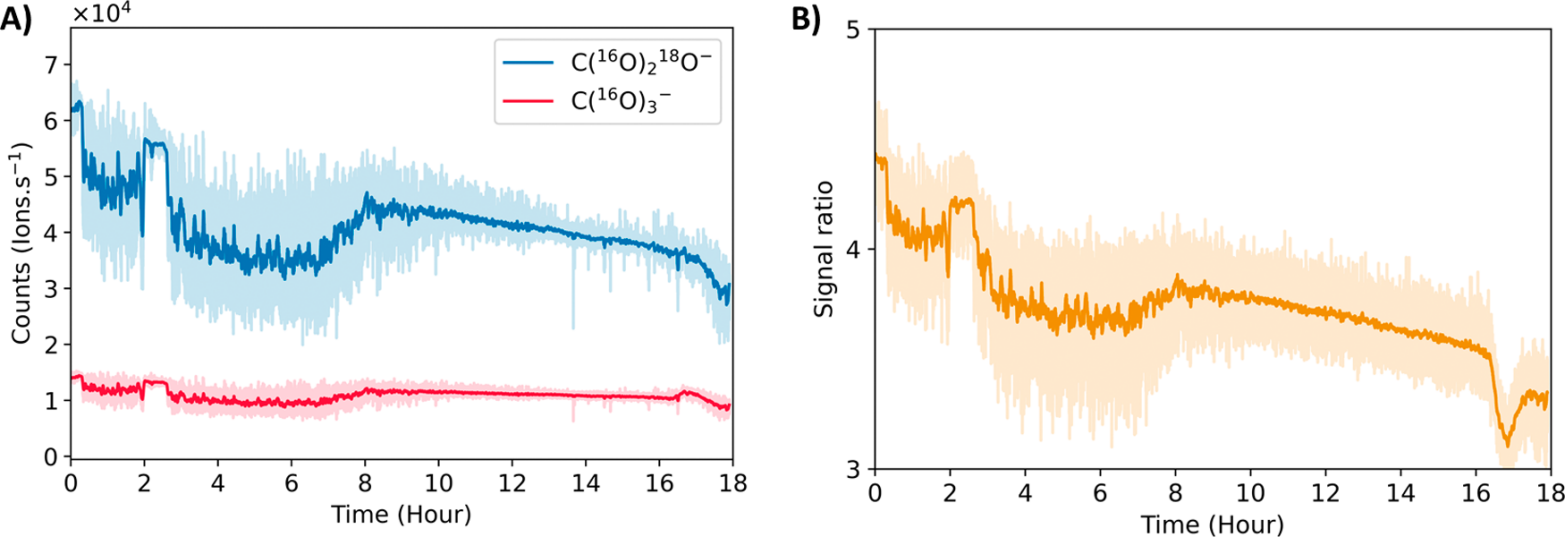
(A) Background subtracted light (blue) and heavy (red) carbonate signals over time. (B) Ratio of heavy to light signals over time. Solid lines represent data averaged over 2 min, and the shaded regions are the 1 Hz data. The main source of variability in the 1 Hz data is the variability of the dilution of sampled O_3_ by room air.

Figure 7 demonstrates that anode corrosion is the primary mechanism of lattice oxygen participation in EOP on NATO, rather than catalytic LOM. Over 18 h, the ratio of the heavy to light carbonate signal decreases from roughly four to three. If AEM were to dominate, no light signal from lattice oxygen would be observed at all. If oxygen from water were to replenish the oxygen vacancies created by the evolution of lattice O_3_, as proposed by LOM,^16,106,107^ a decay in the light signal combined with an increase in the heavy signal over time would be expected. Instead, the decreasing ratio with time suggests that the electrode is irreversibly consumed to make O_3_. The magnitude of the heavy signal is always greater than that of the light signal, which confirms that water is the primary source of O_3_. Furthermore, both signals appear as soon as the reaction is started and remain present until the reaction is stopped, demonstrating that catalysis and corrosion on NATO occur together. However, it is essential to note that these findings neither demonstrate nor refute that lattice corrosion is a prerequisite for EOP and only show that when we generate O_3_, a portion of it is generated through catalyst corrosion.

**Figure 8:**
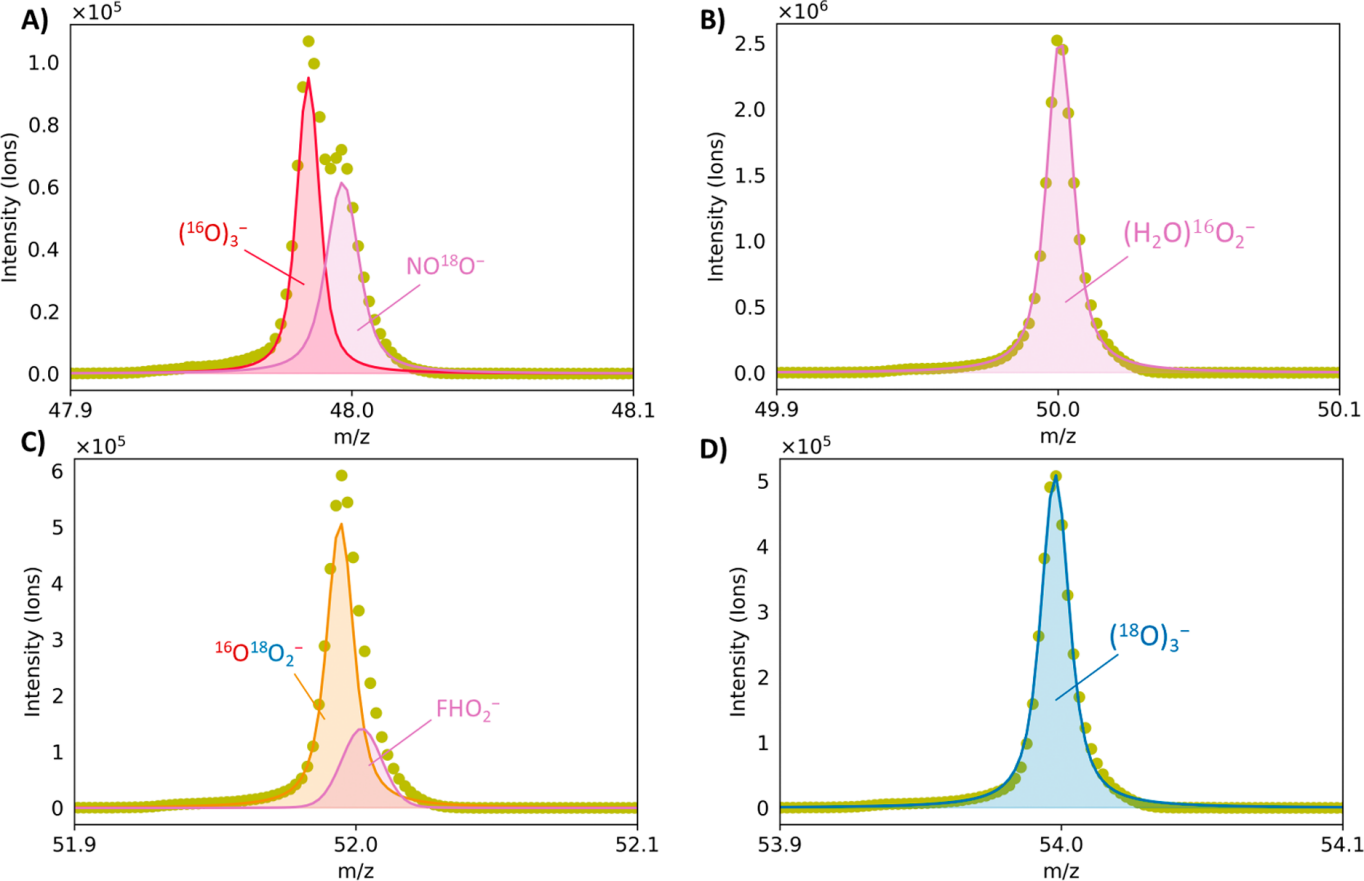
High-resolution mass spectral fitting of O_3_^–^ and overlapping ions at (A) *m*/*z* = 48, (B) *m*/*z* = 50, (C) *m*/*z* = 52, and (D) *m*/*z* = 54.

In addition to the indirect detection of O_3_ through carbonate, it was also detected directly in three different isotopologues, as shown in Figure 8. Ion signals were present in the mass spectrum at the bare O_3_^–^*m*/*z* values, indicating that O_3_ did not react with CO_2_. High-resolution fitting of the acquired mass spectra using the known instrumental line shape shows that O_3_ can be detected with three, one, and zero lattice oxygens. Figure 8 shows representative spectra corresponding to detected ions (O_3_ and overlapping ions) with nominal *m*/*z* values of 48, 50, 52, and 54. The signal at *m*/*z* = 50 predominantly corresponds to an oxygen-water cluster, which prevents the direct detection of O_3_ with two lattice oxygens but does not preclude its existence. Additionally, an overlapping signal from a singly ^18^O substituted nitrogen dioxide ion (N^16^O^18^O^–^) at *m*/*z* = 48 and an ion peak at *m*/*z* = 52 can be detected. The latter peak is likely associated with fluoro hydroperoxide (HFO_2_^–^) resulting from the interaction between reactive species and the fluoropolymer tubing. These findings further demonstrate the participation of lattice oxygen in EOP.

The presence of the “midway” doubly substituted isotopologue ^16^O^18^O_2_ indicates that O_3_ does not evolve directly from the lattice. Given that the formation can occur through a combination of water and lattice oxygen, it is likely that lattice oxygen will corrode into an intermediate compound that can further react to form O_3_. This can be understood by realizing that each isotopologue would require a unique reaction to explain its presence. In addition to our findings, the corrosion of ATO (EOP-inactive) has previously been linked to OER activity.^42^ Combined with our findings, these results suggest that the intermediate compound to which the catalyst corrodes can be converted into both O_3_ and O_2_.

Given that H_2_O_2_ can react to form both O_3_ and O_2_ (as discussed above), the electrochemical corrosion of the catalyst to generate H_2_O_2_ can explain all findings in one reaction
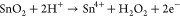
11Notably, under conditions in which the catalyst chemically dissolves, such as ours,^19,31,42^ and H_2_O_2_ is electrochemically formed, direct electrochemical H_2_O_2_ formation via corrosion is thermodynamically satisfied. The chemical dissolution of the catalyst and the viability of generating H_2_O_2_ via corrosion were further investigated using computational quantum chemistry in S1 and S3 Supporting Information. This analysis demonstrates a feasible corrosive pathway that leads to the formation of the O_3_ complex while explaining the lack of electrode stability.

## 4 Conclusions

We have proposed a reaction mechanism for EOP on NATO electrodes that explains observations of free ROS intermediates, the unique role of Ni in the catalyst, and ubiquitous corrosion. Electrochemical analysis suggests the presence of leached Ni^2+^, Ni^3+^, and Ni^4+^. Radical probes demonstrate the existence of ^•^OH and ^•^OOH during EOP, with ^•^OOH being uniquely linked to the production of O_3_ production. The simultaneous emergence of O_3_, ^•^OH, and ^•^OOH at the same potential suggests H_2_O_2_ as a common source for all three species. Computational quantum chemistry calculations support this relationship; both ^•^OH and ^•^OOH can be generated from pseudo-Fenton reactions between H_2_O_2_ and leached Ni cations. Our analysis suggests that the main role of Ni in NATO is catalyzing ^•^OOH from H_2_O_2_ as Ni^4+^, while the main role of Sb is increasing the catalyst conductivity.

Isotopically resolved measurements of O_3_ show that anodic corrosion explains lattice oxygen participation in EOP and that O_3_ is produced in at least three different isotopologues, suggesting that NATO initially corrodes into an intermediate compound before converting into O_3_. A deeper analysis of corrosion, considering the proposed mechanism for EOP, points toward a reaction in which the catalyst corrodes to form H_2_O_2_, which is satisfied thermodynamically. All of the proposed elementary reaction steps involved are shown in Table 3.

**Table 3:** Reactions Involved in EOP on NATO

reaction	description
2H_2_O → H_2_O_2_ + 2H^+^ + 2e^–^	catalytic generation of hydrogen peroxide
SnO_2_ + 4H^+^ → Sn^4+^ + 2H_2_O	chemical dissolution of tin oxide
SnO_2_ + 2H^+^ →Sn^4+^ + H_2_O_2_ + 2e^–^	corrosive generation of hydrogen peroxide
Ni^3+^ → Ni^4+^ + e^–^	oxidation of Ni^3+^to Ni^4+^
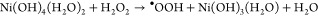	pseudo-Fenton generation of hydroperoxyl radicals
^•^OOH + O^*^ → **O**_3_ + * + **H**^+^ + **e**^–^	oxidation of hydroperoxyl radicals to ozone

Our mechanism is the first to satisfy experimental observations in the EOP on NATO, but it does not provide a definitive relationship between corrosion and catalysis. While the results here show that the two pathways occur together and cause electrode instability, they do not prove that corrosion of the catalyst is required for EOP. Identifying or refuting the existence of such fundamental technological constraints will be critical to any future applications of EOP and other advanced electrochemical oxidation processes.

## Supporting Information Available

Computational treatment of solvation energies for molecular and surface species, assessment of low energy coordination complexes involving Sn^4+^, computational evaluation of pseudo-Fenton reaction energies, computational predictions for the formation of ozone and oxidation of hydroperoxyl radicals, benchmark calculations for the formation of radicals and ozone using various computational quantum chemistry methods, computational predictions for corrosive H_2_O_2_ formation, control electrochemical cyclic voltammetry, carbonate mass spectrum, and XPS spectra (PDF)
