# Supplementary material for: Interplay between Catalyst Corrosion and Homogeneous Reactive Oxygen Species in Electrochemical Ozone Production
Source: ACS Catal. 2024 Apr 18;14(9):6868–80. doi: 10.1021/acscatal.4c01317 (PMC11197020; doi:10.1021/acscatal.4c01317)
Supplement: Supplementary file 1 — cs4c01317_si_001.pdf [file cs4c01317_si_001.pdf]

Supporting Information

Interplay between catalyst corrosion and homogeneous reactive oxygen species in electrochemical ozone production

Rayan Alaufey[a], Lingyan Zhao[b], Andrew Lindsay[c], Tana Siboonruang[a], Qin Wu[d], John A. Keith[b], Ezra Wood[c], Maureen Tang*[a]

[a] Department of Chemical and Biological Engineering, Drexel University, 3101 Chestnut Street, Philadelphia PA, 19104

[b] Department of Chemical and Petroleum Engineering, University of Pittsburgh, 3700 O'Hara Street, Pittsburgh, PA 15261

[c] Department of Chemistry, Drexel University, 3141 Chestnut Street, Philadelphia, PA 19104

[d] Center for Functional Nanomaterials, Brookhaven National Laboratory, Upton, NY, 11973, USA

* Corresponding author: mhtang@drexel.edu

Table of Contents

1. Treatment of solvation energies for molecular and surface species. 3

2. Assessment of low energy coordination complexes involving Sn^{4+} 4

Scheme S1. Thermodynamic cycle for solvated formation energy calculation of potential Sn^{4+} species in aqueous electrolyte. 4

Table S1: Relative formation energies of potential Sn^{4+} species in an electrolyte containing H_2O, $OH^–$, and $SO_4^{2–}$ species. 4

3. Computational evaluation of pseudo-Fenton reaction energies 5

Table S2. Reaction energies of hypothetical pseudo-Fenton processes to form •OH radical involving Ni^{2+}, Ni^{3+}, and Ni^{4+} ions in solution (reactions S1 – S2). 5

Table S3. Reaction energies of hypothetical pseudo-Fenton processes involving Ni^{2+}, Ni^{3+}, and Ni^{4+} ions in solution (reactions S3 – S4)...5

4. Formation of ozone and oxidation of hydroperoxyl radicals 6

Scheme S2. Thermodynamic cycle to calculate reaction energy of ozone formation in aqueous electrolyte. ...6

5. Benchmark calculation of various reactions using different level of theory..............................8

Table S4 reports values for these reactions using PBE calculations using PAW potentials, ORCA calculations using B3LYP calculations, and high-level ab initio thermochemistry calculations using the W1BD method available in Gaussian. This benchmarking indicates that VASP calculations are typically within 0.3 eV of high level W1BD calculations. 8

Table S4. Benchmark of DFT equilibrium potential of various reactions (all data reported in V).......................8

6. H_2O_2 formation via corrosion..9

Scheme S3. Thermodynamic cycle to calculate reaction energy of SnO_2 electrode dissolution in aqueous electrolyte. 9

7. SnO_2 electrode dissolution 9

Table S5. Absolute solvation energies of species reported in the main text and SI (in eV) 9

8. Experimental data 10

Figure S1: Subsequent CV scan for NATO in 0.5 M H_2SO_4AT 75 $mV.s^{-1}$. The scan shows no Ni oxidation features. 10

Figure S2. Representative mass spectrum showing the magnitude of the light (red) and heavy (blue) carbonate signals. 11

Figure S3. A duplicate shorter CIMS measurement (Background subtracted) 12

Figure S4. XPS survey spectra of fresh and used electrodes. 13

Figure S5. XPS survey spectra of the Ni $2p_{3/2}$ region for fresh and used electrodes. 14

References...15

1. Treatment of solvation energies for molecular and surface species.

The solvation energies for molecular species were determined using the SMD continuum solvation model[1] with default parameters for water (dielectric constant = 78.355) as implemented in ORCA.[2,3] For all molecular species except for water, the energy differences between gas and liquid free energies of molecular species are represented as $\Delta G^{*}_{\text{solv}}$, where an additional free energy term reflecting the change in standard states of solutes starting as gases (at 1 bar) and transferring into aqueous states (at 1 M) is included using: $\Delta G^{\circ\rightarrow *} = -RT\ln(V^{*}/V^{\circ})$. This amounts to a correction of +1.89 kcal/mol in addition to the $\Delta G^{*}_{\text{solv}}$ value determined from the SMD solvation model for all solvated molecules except for water. In calculations involving water, we used an additional correction that accounts for the standard state concentration of water being 55.4 M. For more information and discussion see Ref. 4 and cited references therein.[4]

Solvation energies of surfaces had been determined by VASPsol[5,6] calculations with default parameters (dielectric constant = 78.355). No other energy contributions were accounted for with surfaces besides the $\Delta G^{*}_{\text{solv}}$ value since all expressions involved a VASPsol calculation on both sides of the reaction equation, and contributions were thus expected to cancel.

2. Assessment of low energy coordination complexes involving Sn^{4+}

In our modeling of reaction processes involving Sn^{4+} cations in the electrolyte solution, we determined which aqueous phase ligands (H_2O, OH^-, and/or SO_4^{2-}) had the strongest affinity to the Sn^{4+} metal center. Note that the real and absolute solvation energies of Sn^{4+} are not easily known or calculable, but by using the same thermodynamic cycle for all species, this uncertain value cancels out when considering the relative energies between species. Thus, relative stabilities of species can be defined with higher confidence using atomistic thermodynamics[7,8] and the generalized Scheme S1:

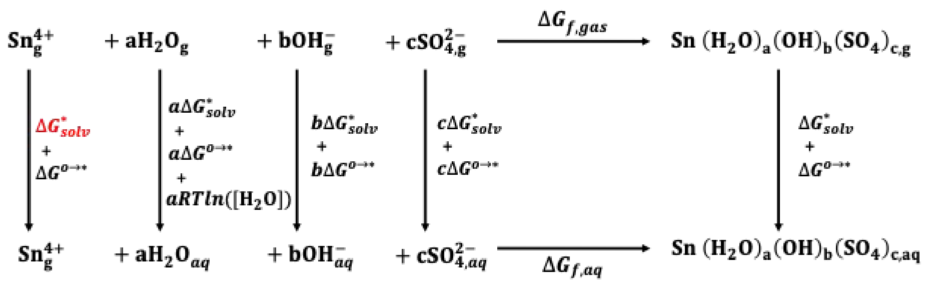

Scheme S1. Thermodynamic cycle for solvated formation energy calculation of potential Sn^{4+} species in aqueous electrolyte.

In our work, we limited ourselves to evaluating just the formation energies of charge neutral species since the continuum solvation energetics are less susceptible to spurious errors. Of the species we considered (see Table S1), $Sn(OH)_4$ was significantly lower than all other species by 1.22 eV and 3.42 eV in gas and aqueous phases, respectively. Our assumption that $Sn(OH)_4$ is the most stable species does not preclude the possibility of other species being more stable, including those having a net charge, but this serves as a starting point for semi-quantitative predictions of redox potentials involving the dissolution of species involving a Sn^{4+} ion.

Table S1: Relative formation energies of potential Sn^{4+} species in an electrolyte containing H_2O, OH^-, and SO_4^{2-} species.

Species	Relative Gas Phase Formation Energy (eV)	Relative Solvated Formation Energy (eV)
$Sn(SO_4)_2$	4.56	8.66
$Sn(SO_4)_2(H_2O)_2$	2.46	7.16
$Sn(SO_4)(OH)_2(H_2O)$	2.00	3.93
$Sn(SO_4)(OH)_2$	1.22	3.42
$Sn(OH)_4$	0	0

3. Computational evaluation of pseudo-Fenton reaction energies

Computational quantum chemistry calculations were performed on four different pseudo-Fenton reactions where we modeled Ni ions as charge neutral species having the form $Ni(OH)_n(H_2O)_{4-n}$ or $Ni(OH)_n(H_2O)_{6-n}$ in the electrolyte, where $n = 2, 3$, and 4 to consider the feasibility of Ni^{2+}, Ni^{3+}, and Ni^{4+} ions in different ligand coordination environments. The reactions shown in Table S2 represent the thermodynamic energies for forming $^{\bullet}OH$ radicals, and this is found to be downhill with an oxidation from a Ni^{2+} to a Ni^{3+} complex, but this is uphill for an oxidation from a Ni^{3+} to a Ni^{4+} complex. Likewise, the reactions shown in Table S3 represent the thermodynamic energies for forming $^{\bullet}OOH$ radicals, and this is found to be uphill for the reduction of a Ni^{3+} complex to a Ni^{2+} complex, but it is significantly downhill for a reduction of a Ni^{4+} complex to a Ni^{3+} complex. While Reaction S4 is significantly downhill, homogenous formation of $Ni(OH)_4$ is expected to be very uphill (via reaction S2) and thus predicted to be unlikely to occur.

Table S2. Reaction energies of hypothetical pseudo-Fenton processes to form $^{\bullet}OH$ radical involving Ni^{2+}, Ni^{3+}, and Ni^{4+} ions in solution (reactions S1 – S2).

Reaction	Reaction energy (eV)
S1. $Ni(OH)_2(H_2O)_2 + H_2O_2 \rightarrow {}^{\bullet}OH + Ni(OH)_3(H_2O) + H_2O$	-0.04
S2. $Ni(OH)_3(H_2O) + H_2O_2 + H_2O \rightarrow {}^{\bullet}OH + Ni(OH)_4(H_2O)_2$	1.71

Table S3. Reaction energies of hypothetical pseudo-Fenton processes involving Ni^{2+}, Ni^{3+}, and Ni^{4+} ions in solution (reactions S3 – S4).

Reaction	Reaction energy (eV)
S3. $Ni(OH)_3\,(H_2O) + H_2O_2 \rightarrow {}^{\bullet}OOH + Ni(OH)_2(H_2O)_2$	0.27
S4. $Ni(OH)_4(H_2O)_2 + H_2O_2 \rightarrow {}^{\bullet}OOH + Ni(OH)_3\,(H_2O) + 2H_2O$	-1.47

4. Formation of ozone and oxidation of hydroperoxyl radicals

Section 3.2 of the main text shows that ozone formation coincides with the formation of $^{\bullet}OOH$ radicals. Since O* is typically expected as a reaction intermediate for water oxidation, and since the $^{\bullet}OOH$ radicals and O* are each 3-e$^-$ and 2-e$^-$ products from water oxidation, we hypothesized that ozone could be formed via a 1-e$^-$ oxidation involving these two species based on the following reaction:

$$OOH_{aq} + O^{*}_{aq} \rightarrow O_{3,aq} + H^{+} + e^{-} + slab_{aq} \quad (S5)$$

The computational hydrogen electrode model was used to predict the redox potential for this process using the thermodynamic cycle in Scheme S2:

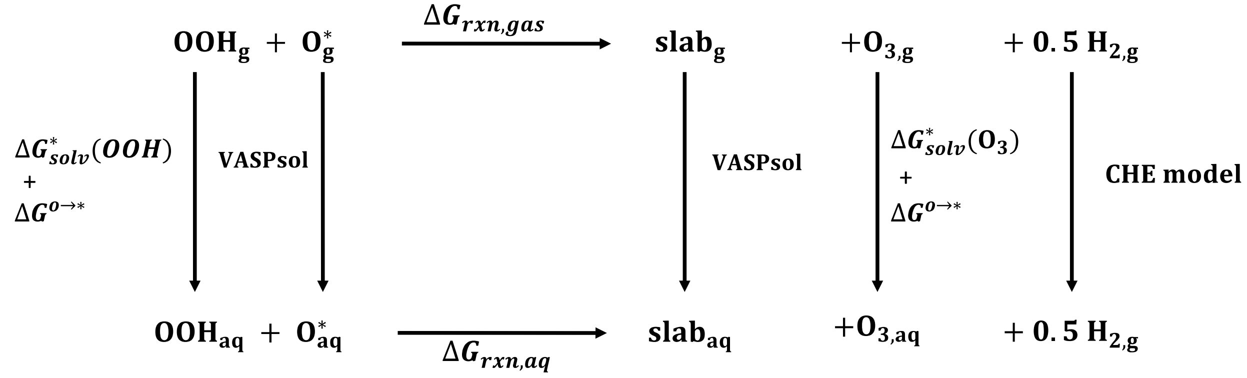

Scheme S2. Thermodynamic cycle to calculate reaction energy of ozone formation in aqueous electrolyte.

The gas phase Gibbs free energy of reaction was calculated to be -0.63 eV. Inclusion of solvation contributions for molecules and slab models causes the reaction energy to be only very slightly downhill with a reaction energy of -0.04 eV. As modeled, the solvation energies of O* using VASPsol and O_3 using the SMD model in Orca had negligibly small contributions (see Table S5 below), signifying that the solvation energies of the clean slab and the $^{\bullet}OOH$ radical have the most impact on the electrochemically relevant reaction energy. While default parameters for VASPsol calculations were used and calculations appeared to converge to reasonable values, we are admittedly skeptical of the physicality of VASPsol calculations run in such a scheme given the lack of experimental data available for benchmarking comparisons.

In recent work in our lab, we have considered the significance of EOP intermediates in the presence of different numbers of explicit water molecules. Thus, we also considered that the

O* and clean slab structures used here might be sensitive to explicit water molecules. These calculations will be discussed in more detail in a future research study, but to summarize a few key points, we found that one explicit water molecule undergoes dissociative adsorption into O* and OH* and results in stabilization energies of -2.45 eV and -1.38 eV on the O* and clean slab structures, respectively. Likewise, a second explicit water molecule results in additional stabilization energies of -0.86 eV and -1.37 eV on these two structures, respectively. Thus, there is clearly a significant energy contribution attributed to explicit solvation of EOP intermediates on tin oxide.

When we consider just the molecular solvation energies using the SMD model and energy contributions from zero, one, and two explicit waters on the surface slab models (and VASPsol calculations are not considered and zero-point energy calculations on the slab models are assumed to be negligibly small), then the electrochemically relevant reaction energy are -0.40eV, +0.67 eV, and +0.17 eV, respectively. In terms of electrochemical reaction potentials, these values correspond to values of -0.40 V, +0.67 V, and +0.17 V since we are modeling a 1e^- oxidation reaction.

Obviously, these are quite different values depending on the number of water molecules used in the model, but we have more confidence in calculations that account for at least one water molecule that others have found dissociates in dynamics studies of tin oxide surfaces.[9] Also, since the $^{\bullet}OOH$ radicals are experimentally observed to form at potentials of about 1.7 V (theoretical value = 1.72 V, see below), since our experimentally observed onset of O_3 detection occurs at 2.15 V, and since the O* intermediate is assumed to be a non-controversial intermediate in water oxidation and EOP reaction mechanisms, there should be an electrochemical reaction that generates O_3 and requires a potential of 2.15 V – 1.7 V = 0.45 V relative to that for a state involving the $^{\bullet}OOH$ radical and O* intermediate. We deduce that Reaction S5 is a reasonable process for this role because oxidation potentials involving one and two explicit water molecules on the slab model surface are modeled as +0.67 V and +0.17 V, which happen to both straddle the +0.45 V value. Thus, we propose that EOP will involve a process resembling Equation S5 until more sophisticated experiments and/or calculations can provide more insight.

5. Benchmark calculation of various reactions using different level of theory

We assessed the accuracy of various computational quantum chemistry methods in calculating electronic energies based on five electrochemical reactions that involved EOP intermediates:

$$H_2O \rightarrow \ ^{\bullet}OH^{\cdot} + H^+ + e^- \#(1)$$

$$2H_2O \rightarrow \ H_2O_2 + 2H^+ + 2e^- \#(2)$$

$$2H_2O \rightarrow \ ^{\bullet}OOH + 3H^+ + 3e^- \#(3)$$

$$H_2O_2 \rightarrow \ ^{\bullet}OOH^{\cdot} + H^+ + e^- \#(4)$$

$$^{\bullet}OOH + \frac{1}{2}O_2 \rightarrow O_3 + \ H^+ + e^- \ \#(5)$$

Table S4 reports values for these reactions using PBE calculations using PAW potentials, ORCA calculations using B3LYP calculations, and high-level ab initio thermochemistry calculations using the W1BD method available in Gaussian. This benchmarking indicates that VASP calculations are typically within 0.3 eV of high level W1BD calculations.

Table S4. Benchmark of DFT equilibrium potential of various reactions (all data reported in V).

Reaction number	Available thermodynamic data	VASP-PBE: gas	VASP-PBE: solvated	ORCA-B3LYP: gas	ORCA-B3LYP: solvated	Gaussian W1BD-gas	Gaussian W1BD-solvated
**1**	2.2-2.73	2.96	2.97	2.50	2.51	2.74	2.75
**2**	1.76	1.71	1.73	1.68	1.70	1.85	1.87
**3**	1.70	1.52	1.56	1.47	1.51	1.68	1.72
**4**	1.58	1.13	1.21	1.05	1.13	1.33	1.41
**5**	N/A	1.31	1.31	1.89	1.88	1.58	1.58

Note that all cases used the same free energy corrections (using ideal gas, rigid rotor, and harmonic oscillator approximations) from ORCA B3LYP calculations. All solvated reaction energies made use of the same solvation energies calculated from ORCA B3LYP calculations.

6. H_2O_2 formation via corrosion

Using Scheme S3, we have modeled water oxidation to H_2O_2 coupled with a corrosion process that results in the formation of the Sn^{4+} cation, which according to data in Table S1 is most stable as a $Sn(OH)_4$ complex. The electrochemical potential for this process is modeled as 3.02 V and 3.36 V when accounting for one and two explicit waters dissociated on each of the two surface models for SnO_2, respectively. Given that individual water molecules provide significantly large stabilization energies (see Section 4 of the SI above), we expect these values indicate that our model is simply not adequate for the true process that should occur at steady state potentials less than 2.6 V. We note that modeling structural defects at surfaces within a solid/liquid interface are very challenging and likely would require dynamics simulations with accurate atomistic potentials.

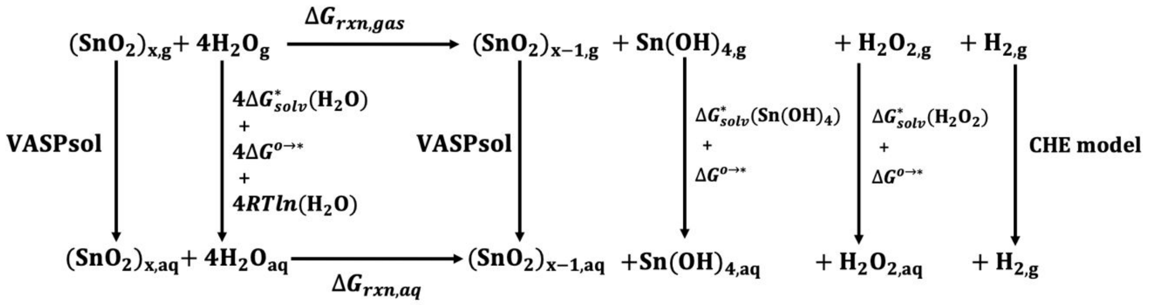

Scheme S3. Thermodynamic cycle to calculate reaction energy of SnO_2 electrode dissolution in aqueous electrolyte.

7. SnO_2 electrode dissolution

Table S5 shows calculated solvation energies obtained using the SMD model as implemented in ORCA.

Table S5. Absolute solvation energies of species reported in the main text and SI (in eV)

H_2O	$Sn(OH)_4$	H_2O_2	OOH	O_3
-0.17	-0.81	-0.30	-0.23	-0.06

8. Experimental data

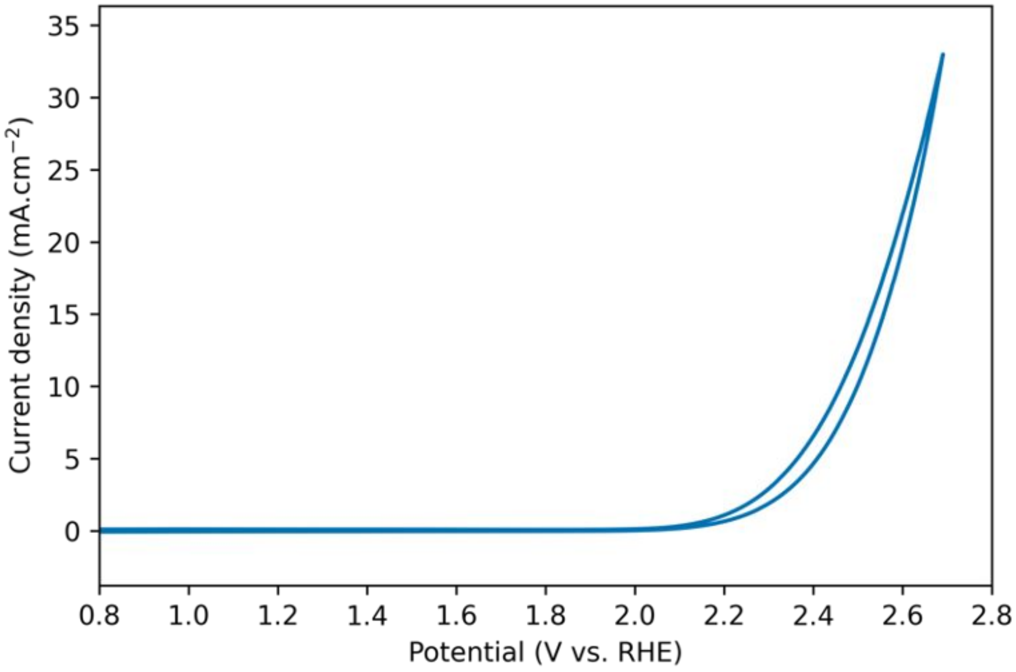

***Figure* S*1: Subsequent CV scan for NATO in 0.5 M H_2SO_4AT* 75 mV.s^{-1}. The scan shows no Ni oxidation features.**

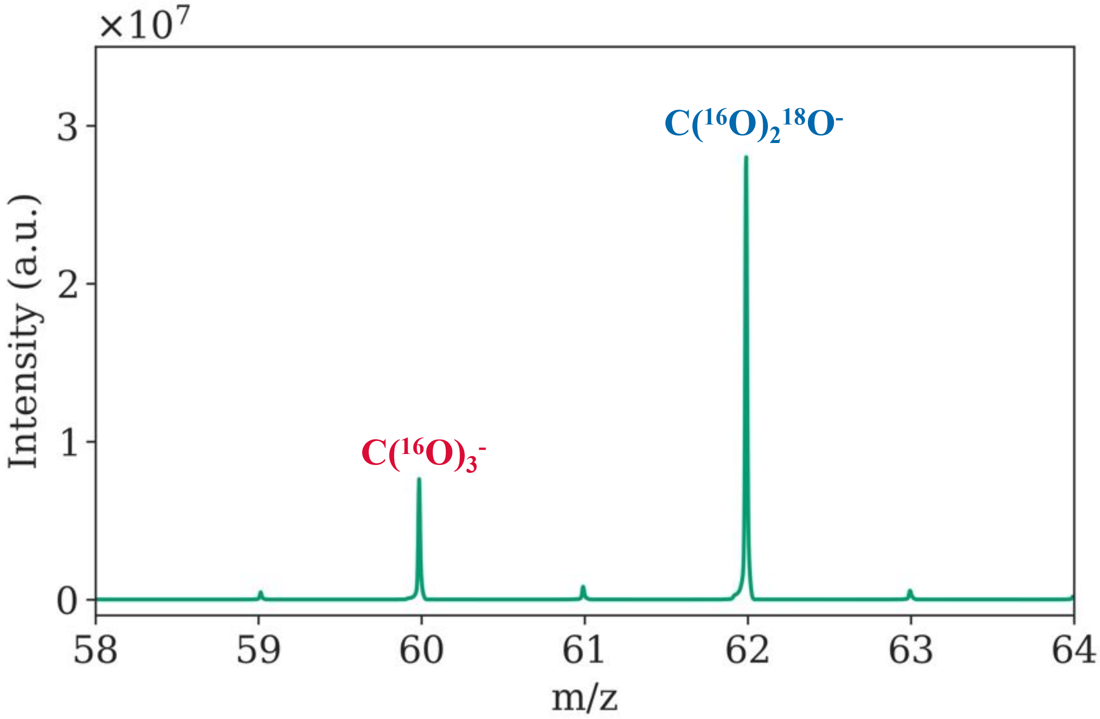

Figure **S2. Representative mass spectrum showing the magnitude of the light (red) and heavy (blue) carbonate signals.**

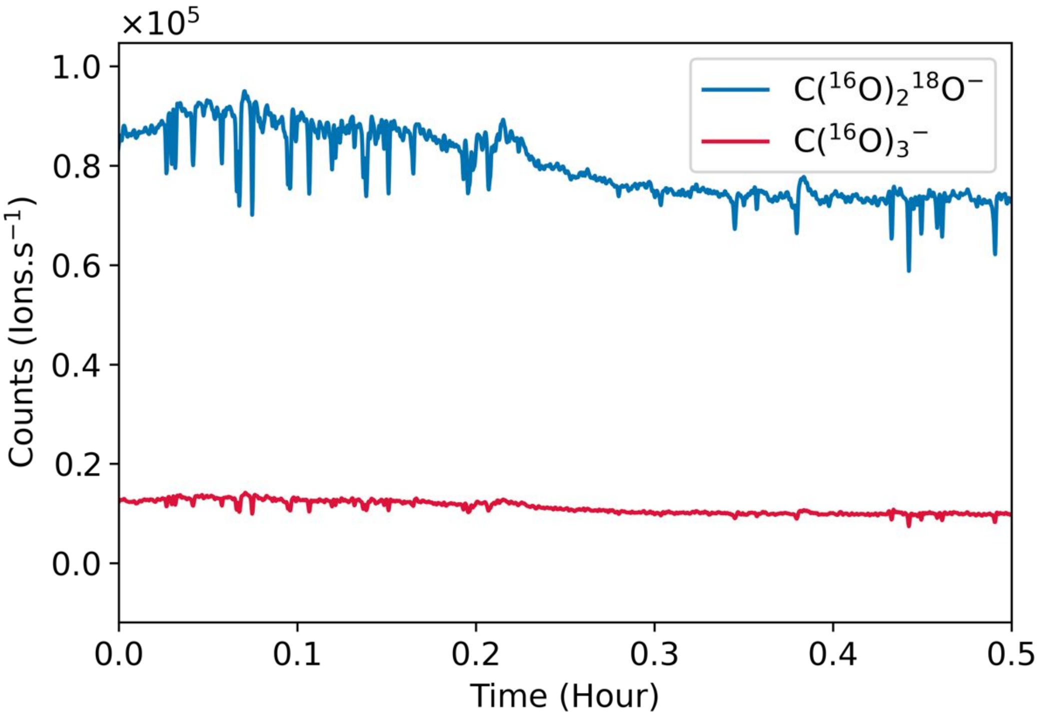

***Figure* S*3.* A duplicate shorter CIMS measurement (Background subtracted)**

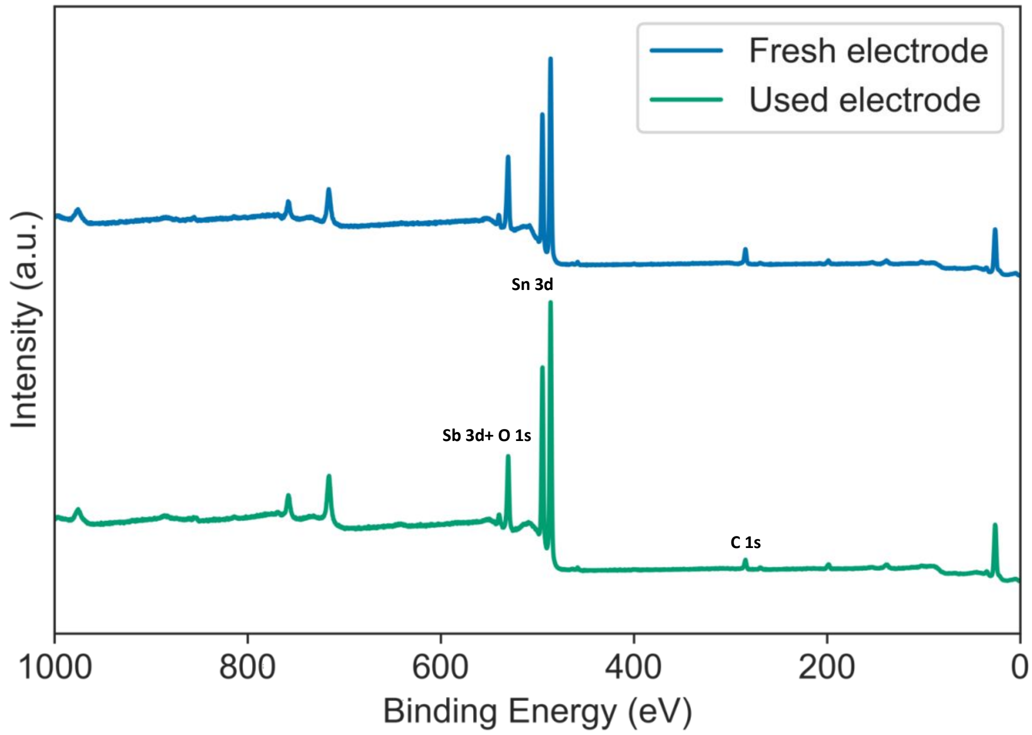

***Figure* S*4.* XPS survey spectra of fresh and used electrodes.**

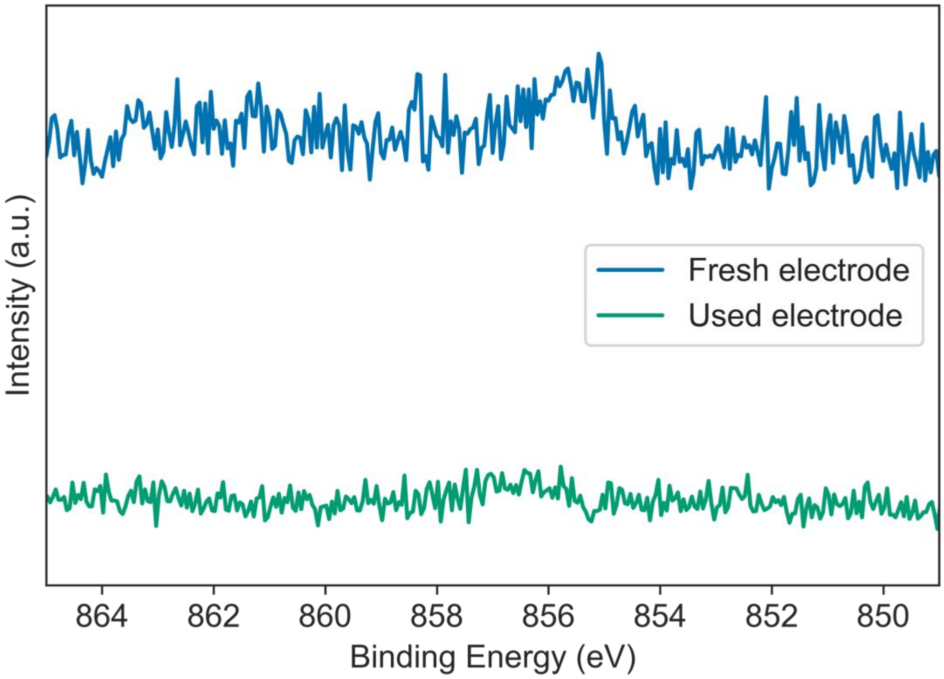

***Figure* S5. XPS survey spectra of the Ni $2p_{3/2}$ region for fresh and used electrodes.**

References

(1) Marenich, A. V.; Cramer, C. J.; Truhlar, D. G. Universal Solvation Model Based on Solute Electron Density and on a Continuum Model of the Solvent Defined by the Bulk Dielectric Constant and Atomic Surface Tensions. *Journal of Physical Chemistry B* **2009**, *113* (18), 6378–6396. https://doi.org/10.1021/JP810292N/SUPPL_FILE/JP810292N_SI_003.PDF.

(2) Neese, F. The ORCA Program System. *Wiley Interdiscip Rev Comput Mol Sci* **2012**, *2* (1), 73–78. https://doi.org/10.1002/WCMS.81.

(3) Neese, F. Software Update: The ORCA Program System, Version 4.0. *Wiley Interdiscip Rev Comput Mol Sci* **2018**, *8* (1). https://doi.org/10.1002/WCMS.1327.

(4) Keith, J. A.; Carter, E. A. Quantum Chemical Benchmarking, Validation, and Prediction of Acidity Constants for Substituted Pyridinium Ions and Pyridinyl Radicals. *J Chem Theory Comput* **2012**, *8* (9), 3187–3206. https://doi.org/10.1021/CT300295G/SUPPL_FILE/CT300295G_SI_001.PDF.

(5) Mathew, K.; Sundararaman, R.; Letchworth-Weaver, K.; Arias, T. A.; Hennig, R. G. Implicit Solvation Model for Density-Functional Study of Nanocrystal Surfaces and Reaction Pathways. *Journal of Chemical Physics* **2014**, *140* (8). https://doi.org/10.1063/1.4865107/1003843.

(6) Mathew, K.; Kolluru, V. S. C.; Mula, S.; Steinmann, S. N.; Hennig, R. G. Implicit Self-Consistent Electrolyte Model in Plane-Wave Density-Functional Theory. *Journal of Chemical Physics* **2019**, *151* (23), 234101. https://doi.org/10.1063/1.5132354/15566182/234101_1_ACCEPTED_MANUSCRIPT.PDF.

(7) Griego, C. D.; Maldonado, A. M.; Zhao, L.; Zulueta, B.; Gentry, B. M.; Lipsman, E.; Choi, T. H.; Keith, J. A. Computationally Guided Searches for Efficient Catalysts through Chemical/Materials Space: Progress and Outlook. *The Journal of Physical Chemistry C* **2021**, *125* (12), 6495–6507. https://doi.org/10.1021/acs.jpcc.0c11345.

(8) Reuter, K.; Scheffler, M. First-Principles Atomistic Thermodynamics for Oxidation Catalysis: Surface Phase Diagrams and Catalytically Interesting Regions. *Phys Rev Lett* **2003**, *90* (4), 4. https://doi.org/10.1103/PHYSREVLETT.90.046103/FIGURES/2/MEDIUM.

(9) Santarossa, G.; Hahn, K.; Baiker, A. Free Energy and Electronic Properties of Water Adsorption on the SnO 2(110) Surface. *Langmuir* **2013**, *29* (18), 5487–5499. https://doi.org/10.1021/LA400313A.
